# Community-Acquired Pneumonia in the Immunocompromised Host: Epidemiology and Outcomes

**DOI:** 10.1093/ofid/ofad565

**Published:** 2023-11-22

**Authors:** Julio A Ramirez, Thomas R Chandler, Stephen P Furmanek, Ruth Carrico, Ashley M Wilde, Daniya Sheikh, Raghava Ambadapoodi, Vidyulata Salunkhe, Mohammad Tahboub, Forest W Arnold, Jose Bordon, Rodrigo Cavallazzi, Louisville CAP in the Immunocompromised Study Group, Mohammed Khalid Abdulaziz Abbas, Ahmed Abdelhaleem, Aisha Olanike Adigun, Usman Ali Akbar, Oluwasegun Akinti, Ahmed Ali, Raghava Sekhar Ambadapoodi, Javaria Anwer, Saman Bahram, Aditya Bamboria, Laxman Bhandari, FNU Deepti, Joanna Ekabua, Sheref Abdelgawad Hassan Elseidy, Aiman Fatima, Farwah Fatima, Shivam Gulati, Syed Hassan, Shriya Khurana, Shameera Masthan, Rehab Salah Mohamed, Vivek Soorya Sathya Moorthy, Amal Mumtaz, Aleena Naeem, Keerthi Poladi, Lucia Puga Sanchez, Adnan Qureshi, Prasanna Raut, Vidyulata Salunkhe, Harideep Samanapally, Balaji Srinivasa Sekaran, Syed Zain Shah, Daniya Sheikh, Mohammad Tahboub, Rupalakshmi Vijayan, Mounica Vorla, Sudeep Yadav, Zarlakhta Zamani

**Affiliations:** Norton Infectious Diseases Institute, Norton Healthcare, Louisville, Kentucky, USA; Division of Infectious Diseases, School of Medicine, University of Louisville, Louisville, Kentucky, USA; Norton Infectious Diseases Institute, Norton Healthcare, Louisville, Kentucky, USA; Norton Infectious Diseases Institute, Norton Healthcare, Louisville, Kentucky, USA; Norton Infectious Diseases Institute, Norton Healthcare, Louisville, Kentucky, USA; Norton Infectious Diseases Institute, Norton Healthcare, Louisville, Kentucky, USA; Division of Infectious Diseases, School of Medicine, University of Louisville, Louisville, Kentucky, USA; Division of Infectious Diseases, School of Medicine, University of Louisville, Louisville, Kentucky, USA; Division of Infectious Diseases, School of Medicine, University of Louisville, Louisville, Kentucky, USA; Division of Infectious Diseases, School of Medicine, University of Louisville, Louisville, Kentucky, USA; Division of Infectious Diseases, School of Medicine, University of Louisville, Louisville, Kentucky, USA; Washington Health Institute, Washington, DC, USA; Division of Pulmonary, Critical Care and Sleep Disorders, School of Medicine, University of Louisville, Louisville, Kentucky, USA

## Abstract

**Background:**

The epidemiology and outcomes of community-acquired pneumonia (CAP) in immunocompromised hosts (ICHs) are not well defined. The objective of this study was to define the epidemiology and outcomes of CAP in ICHs as compared with non-ICHs.

**Methods:**

This ancillary study included a prospective cohort of hospitalized adult Louisville residents with CAP from 1 June 2014 to 31 May 2016. An ICH was defined per the criteria of the Centers for Disease Control and Prevention. Geospatial epidemiology explored associations between ICHs hospitalized with CAP and income level, race, and age. Mortality for ICHs and non-ICHs was evaluated during hospitalization and 30 days, 6 months, and 1 year after hospitalization.

**Results:**

A total of 761 (10%) ICHs were identified among 7449 patients hospitalized with CAP. The most common immunocompromising medical conditions or treatments were advanced-stage cancer (53%), cancer chemotherapy (23%), and corticosteroid use (20%). Clusters of ICHs hospitalized with CAP were found in areas associated with low-income and Black or African American populations. Mortality by time point for ICHs vs non-ICHs was as follows: hospitalization, 9% vs 5%; 30 days, 24% vs 11%; 6 months, 44% vs 21%; and 1 year, 53% vs 27%, respectively.

**Conclusions:**

Approximately 1 in 10 hospitalized patients with CAP is immunocompromised, with advanced-stage cancer being the most frequent immunocompromising condition, as seen in half of all patients who are immunocompromised. Risk for hospitalization may be influenced by socioeconomic disparities and/or race. ICHs have a 2-fold increase in mortality as compared with non-ICHs.

## Teaser

Approximately 1 in 10 patients hospitalized with community-acquired pneumonia is immunocompromised. Socioeconomic factors may influence risk for hospitalization. Immunocompromised hosts hospitalized with community-acquired pneumonia have a 2-fold increase in mortality as compared with nonimmunocompromised hosts.

The Centers for Disease Control and Prevention (CDC) estimated in 2013 that approximately 3% of the adult population of the United States were immunocompromised [1]. Today, the prevalence of immunocompromised hosts (ICHs) in the United States is likely to be significantly higher due to the increased use of biological immune modulators as therapy for a broad range of rheumatologic, dermatologic, gastrointestinal, and autoimmune diseases [2]. Additionally, the improved survival of patients with cancer, as well as the longer survival of recipients of organ transplants, increases the prevalence of ICHs in the United States [3].

Community-acquired pneumonia (CAP) is the primary infection requiring hospital care for adults in the United States and is the primary cause of death attributed to infection [4, 5]. Studies have shown that patients hospitalized with CAP have some abnormality in the immune system primarily due to advanced age (immunosenescence) or the presence of comorbidities such as chronic obstructive pulmonary disease, heart failure, and diabetes [6, 7]. Patients who are immunocompromised have medical illnesses or treatments that severely compromise their immune function, placing them at increased risk of acquiring CAP due to low virulence or opportunistic pathogens [8]. The CDC recently conducted a systematic review to establish the underlying conditions predisposing patients with SARS-CoV-2 infection to poor clinical outcomes such as hospitalization, admission to the intensive care unit, mechanical ventilation, or death. In this study, ICHs face an elevated risk of poor outcomes due to SARS-CoV-2, as do patients without immunocompromising conditions but with specific medical comorbidities [9].

Even though ICHs are at increased risk for hospitalization and poor outcomes due to respiratory infections, there is a paucity of data evaluating the epidemiology and clinical outcomes of ICHs hospitalized with CAP. The objective of this study was to define the epidemiology and clinical outcomes of ICHs hospitalized with CAP in the city of Louisville, Kentucky.

## METHODS

### Study Design and Patients

This was an ancillary study of a prospective population-based cohort study of consecutive hospitalized adult (age ≥18 years) Louisville residents with CAP. The parent study took place at all adult hospitals in Louisville from 1 June 2014 to 31 May 2016. Data from the parent cohort study were previously published [7]. For this ancillary study, all medical records of hospitalized patients with CAP from the parent study were reviewed to define the presence of immunocompromising medical illnesses or treatments.

### Immunocompromising Conditions

Based on the CDC definition of individuals who are immunocompromised [10], the following medical illnesses or treatments were used to define a patient as immunocompromised: primary immunodeficiency disease, advanced-stage cancer (stage III or IV solid cancer or hematologic cancer, as defined in the supplementary materials), advanced HIV infection (CD4 T-lymphocyte count <200 cells/mL or <14%), solid organ transplantation, hematopoietic stem cell transplantation, cancer chemotherapy, biological immune modulators, corticosteroid therapy with a ≥20-mg dose of prednisone or equivalent daily for at least 14 days prior to hospitalization, or disease-modifying antirheumatic drugs.

### Classification of Patients According to Function of the Immune System

Considering the function of the immune system, patients with CAP were categorized into 4 ordinal groups: without identifiable immunologic abnormality, abnormal immune system, immunocompromised, or severely immunocompromised. Patients without identifiable immunologic abnormality were aged <65 years with no comorbidities or immunocompromising conditions. Patients with an abnormal immune system were aged ≥65 years or any age with at least 1 of the following comorbidities but none of the immunocompromising conditions: chronic obstructive pulmonary disease, heart failure, cerebrovascular disease, diabetes, renal disease, liver disease, or obesity. The immunocompromised cohort consisted of adult patients of any age with 1 immunocompromising condition, and the severely immunocompromised cohort consisted of adult patients of any age with >1 immunocompromising condition. Patients without identifiable immunologic abnormality and patients with an abnormal immune system included all nonimmunocompromised adults (ie, non-ICHs). Patients who were immunocompromised and severely immunocompromised included all ICHs.

### Geospatial Epidemiology

The geomasked location of the home address of each ICH who enrolled in the study was obtained through the US Census Bureau website [11]. A LISA map (ie, local indicators of spatial association) [12] was produced to identify clusters of high and low incidence of patients who were immunocompromised and hospitalized due to CAP at the census tract level. The Kulldorff spatial scan statistic [13, 14] was used to calculate significant areas of risk for hospitalization attributed to CAP, accounting for the underlying population density. A kernel density heat map was created by using each unique patient's geomasked home location at the time of first hospitalization, with the area of increased risk determined by the Kulldorff spatial scan statistic overlaid. Kernel density maps were created to compare the spatial distribution of ICHs hospitalized owing to CAP to census tract–level estimates of poverty, race, and age. A complete description of the geospatial methods is available in the supplementary materials.

### Pneumonia Severity Scores

The Pneumonia Severity Index (PSI) [15] and the CURB-65 [16] were used to evaluate patients by their initial presentation to the hospital. Patients were considered to have a high PSI score if they were risk class IV or V, and a CURB-65 score of 3, 4, or 5 was considered high. This was compared between ICHs and non-ICHs. Pneumonia severity scores were also evaluated by immunocompromising condition.

### Cardiovascular Events

A patient was defined as having a cardiovascular event if any of the following were reported during hospitalization: new arrhythmia, acute worsening of long-term arrhythmia, pulmonary edema, pulmonary embolism, acute myocardial infarction, or stroke. Rates of cardiovascular events were compared between ICHs and non-ICHs. Cardiovascular events were also compared by immunocompromising condition.

### Etiology of CAP

Microbiological workup by standard of care was recorded. An organism identified by sputum cultures, blood cultures, respiratory sample polymerase chain reaction, or urinary antigen detection was considered the etiology of CAP. Etiologies were characterized between ICHs and non-ICHs.

### Time to Clinical Stability and Length of Stay

Time to clinical stability was defined as the first day that a patient met the following 3 criteria: afebrile for at least 8 hours, improvement in cough and/or shortness of breath, and normalization of white blood cell count. Hospitalized length of stay was calculated as the number of days from admission to discharge. Time to clinical stability and length of stay were compared between ICHs and non-ICHs.

### Rehospitalization due to CAP

Rehospitalization due to CAP in the following year was assessed only for patients enrolled during the first year of the study and alive at hospital discharge. The rate of rehospitalization was compared between ICHs and non-ICHs. Rehospitalization for CAP was also evaluated by immunocompromising condition.

### Mortality

All-cause mortality for all ICHs and non-ICHs with CAP was evaluated during hospitalization and at 30 days, 6 months, and 1 year after hospitalization. After discharge, mortality was evaluated by reviewing medical records and by matching patients’ social security numbers with mortality data obtained from the Kentucky Department for Public Health’s Office of Vital Statistics. A post hoc analysis was performed comparing ICHs who had advanced-stage lung cancer, corticosteroid use, and advanced HIV with other ICHs and non-ICHs to evaluate these immunocompromising conditions’ impact on mortality.

### Human Subjects Protection

The study was approved by the Institutional Review Board at the University of Louisville Human Subjects Research Protection Program Office (11.0613) and by the research office at each participating hospital. The study was exempt from informed consent.

### Study Coordinating Center

This study coordinating center, located at the University of Louisville’s Division of Infectious Diseases, directed all operational and data aspects of the study. The Norton Infectious Diseases Institute performed data processing and analysis.

### Statistical Analysis

Continuous data were presented as median and IQR, and categorical data were presented as frequency and percentage. Comparisons between ICHs and non-ICHs were tested with Mann-Whitney *U* tests for continuous data and chi-square tests of independence for categorical data. Kaplan-Meier estimation was performed for time-to-event outcomes and compared with log-rank tests. *P* values <.05 were considered statistically significant. All statistical analysis was performed with R version 4.2.2 [17].

## RESULTS

### Study Population

**Figure 1.**
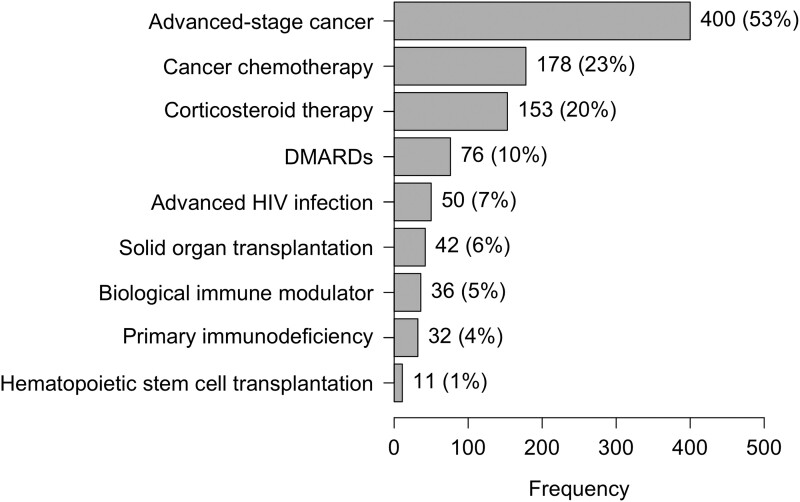
Distribution of immunocompromising medical conditions and treatments. Conditions and treatments are not mutually exclusive; percentages add to >100%. DMARD, disease-modifying antirheumatic drug.

**Figure 2.**
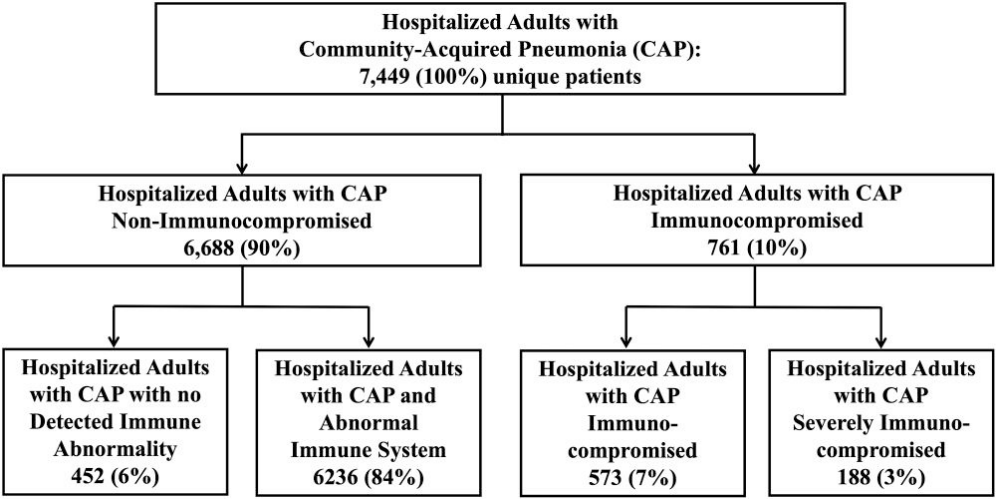
Distribution of patients hospitalized with community-acquired pneumonia by immune function.

A total of 761 (10%) ICHs were identified from 7449 unique patients in the parent study. The immunocompromising conditions of ICHs are depicted in Figure 1. The most common immunocompromising condition was advanced-stage cancer in 400 patients (53%); among these, lung cancer was the most prevalent in 181 (45%) patients. When patients were classified according to function of the immune system, 452 (6%) were without identifiable immunologic abnormality, 6236 (84%) had an abnormal immune system, 573 (7%) were immunocompromised, and 188 (3%) were severely immunocompromised. Figure 2 outlines the study population according to function of the immune system. A description of patients with >1 immunocompromising condition is presented in Supplementary Table 1.

Patient demographics and comorbidities comparing ICHs and non-ICHs are provided in Table 1. When compared with non-ICHs, the median age for ICHs was younger (65 vs 68, *P* < .001), and fewer ICHs were nursing home residents (9% vs 14%, *P* = .001). There were no differences in sex and race between ICH and non-ICHs. A higher proportion of ICHs were former smokers (43% vs 36%, *P* < .001) and had histories of noncirrhotic liver disease (10% vs 7%, *P* = .001). Histories of atrial fibrillation, diabetes mellitus, cerebrovascular disease, obesity, coronary artery disease, hypertension, and hyperlipidemia were significantly more frequent among non-ICHs. Physical examination findings and laboratory values, as well as minor and major criteria for severe CAP per the American Thoracic Society/Infectious Diseases Society of America [18], are outlined in Supplementary Table 2.

**Table 1.** Patient Characteristics of ICHs vs Non-ICHs

	Patients, No. (%)		
	ICH (n = 761)	Non-ICH (n = 6688)	*P* Value^a^
Demographics and social history			
Age, y, median [IQR]	65 [57–76]	68 [56–80]	<.001^b^
Sex: male	356 (47)	3087 (46)	.773
Nursing home resident	72 (9)	915 (14)	.001
Black	167 (22)	1308 (20)	.129
Former smoker	329 (43)	2431 (36)	<.001
History of comorbid disease			
Obesity	201 (26)	2414 (36)	<.001
Diabetes	208 (27)	2225 (33)	.001
Renal disease	200 (26)	1985 (30)	.056
Chronic obstructive pulmonary disease	338 (44)	3137 (47)	.205
Liver disease	76 (10)	452 (7)	.001
Cerebrovascular disease	72 (9)	883 (13)	.004
Coronary artery disease	184 (24)	2034 (30)	<.001
Hypertension	475 (62)	4678 (70)	<.001
Hyperlipidemia	278 (37)	2956 (44)	<.001
Prior myocardial infarction	80 (11)	832 (12)	.139
Atrial fibrillation	120 (16)	1345 (20)	.005

Abbreviation: ICH, immunocompromised host.

^a^Chi-square test of independence unless otherwise noted.

^b^Mann-Whitney *U* test.

### Geospatial Epidemiology

**Figure 3.**
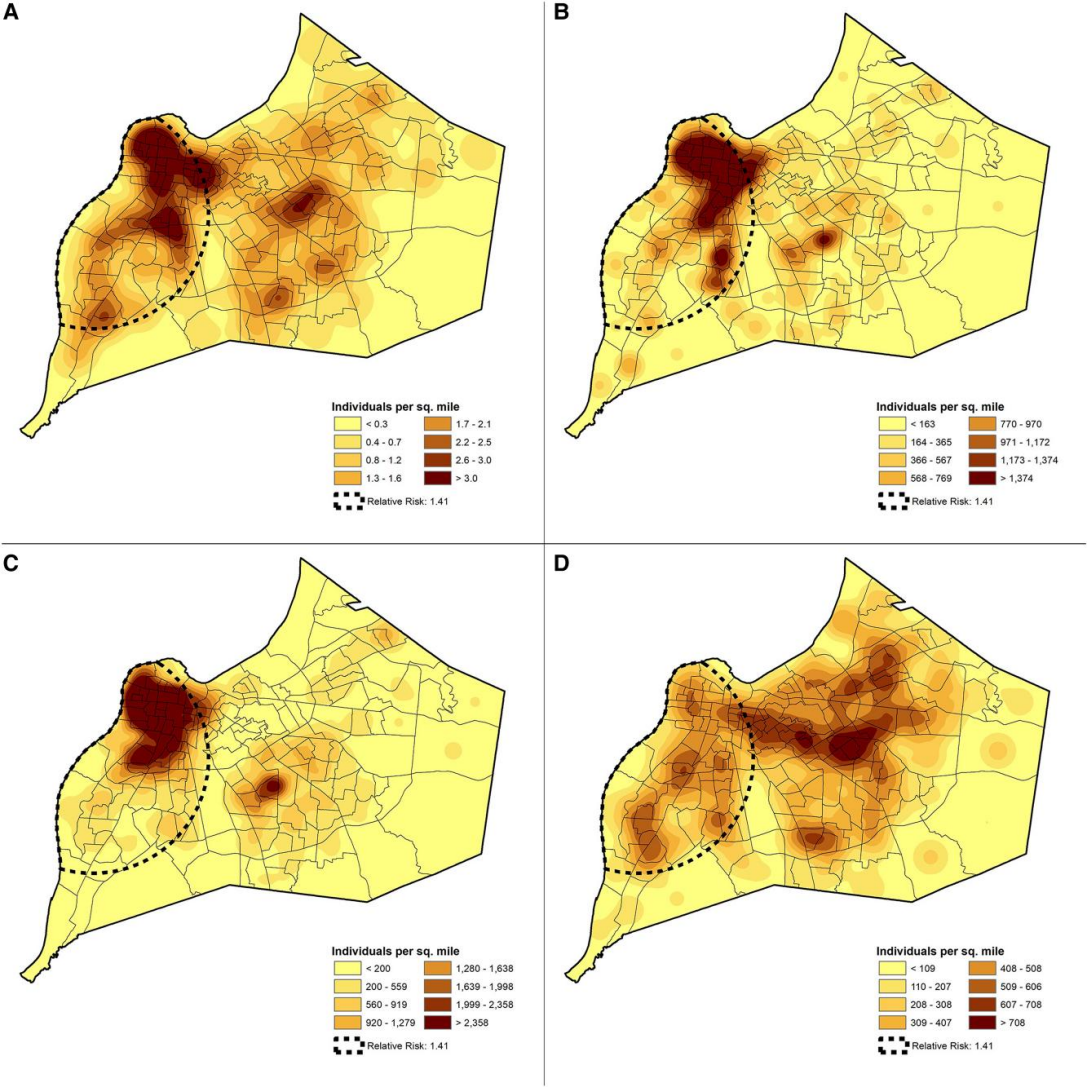
Distribution of immunocompromised hosts in Louisville, Kentucky, vs poverty, race, and age. *A*, Hospitalized cases of immunocompromised hosts with community-acquired pneumonia in Jefferson County, Kentucky, with an area of an increased risk of hospitalization outlined by dotted line. Publicly available census data collected from the same area and year quantifying the number individuals (*B*) at or below the national poverty level, (*C*) of Black or African American race, and (*D*) of advanced age. Area of relative risk was identified through the Kulldorff spatial scan statistic.^13,14^

Supplementary Figure 1 depicts the LISA map of Louisville showing clusters of high rates of ICHs hospitalized due to CAP in census tracts located in the northwestern section of the city. The heat map of ICHs with CAP in the city of Louisville is illustrated in Figure 3*A*. A zone of high risk for hospitalization due to CAP was identified in the western section of the city (risk ratio, 1.41; *P* = .002), overlapping the same area on the LISA map. The clustering of ICHs with CAP in the western section of the city overlaps with the census tracts where the average population has an annual income below the national poverty level (Figure 3*B*) and is of Black or African American race (Figure 3*C*). Census tracts with the highest percentage of elderly population were in the eastern section of the city (Figure 3*D*).

### Pneumonia Severity Scores

A total of 569 (75%) ICHs had PSI risk class IV or V, as opposed to 3947 (59%) non-ICHs (*P* < .001). According to CURB-65, 257 (34%) ICHs had scores ≥3 vs 2439 (36%) non-ICHs (*P* = .135). Pneumonia severity scores by immunocompromising conditions are provided in Supplementary Table 3.

### Cardiovascular Events

A total of 47 (6%) ICHs experienced a cardiovascular event during hospitalization, as opposed to 520 (8%) non-ICHs (*P* = .133). The most frequent cardiovascular event was new arrhythmia in ICHs and non-ICHs. The frequencies of cardiovascular events are provided in Supplementary Table 4.

### Etiology of CAP

Supplementary Table 5 depicts the microbiological workup of the patient cohort. The etiology of CAP was identified in 24% of ICHs and 23% of non-ICHs. The most common etiology identified for ICHs and non-ICHs was *Streptococcus pneumoniae* in 40% of ICHs and 41% of non-ICHs, followed by methicillin-resistant *Staphylococcus aureus* in 9% of ICHs and 10% of non-ICHs. A complete list of etiologies is presented in Table 2.

**Table 2.** Distribution of Identified Microorganisms: ICH vs Non-ICH

	Patients,^a^ No. (%)	
	ICH (n = 761)	Non-ICH (n = 6688)
Total with identified pathogen	180 (24)	1519 (23)
Etiology among patients with identified pathogen		
*Streptococcus pneumoniae*	72 (40)	619 (41)
Methicillin-resistant *Staphylococcus aureus*	17 (9)	157 (10)
*Pseudomonas aeruginosa*	13 (7)	66 (4)
Methicillin-susceptible *Staphylococcus aureus*	12 (7)	89 (6)
*Staphylococcus* other	9 (5)	53 (4)
*Escherichia coli*	8 (4)	34 (2)
Rhinovirus/enterovirus	8 (4)	116 (8)
*Aspergillus* spp	8 (4)	2 (<1)
*Haemophilus influenzae*	7 (4)	66 (4)
*Klebsiella pneumoniae*	6 (3)	33 (2)
*Streptococcus* other	5 (3)	43 (3)
Metapneumovirus	4 (2)	52 (4)
Parainfluenza	4 (2)	23 (2)
Corona virus	3 (2)	28 (2)
*Acinetobacter* spp	2 (1)	13 (1)
*Streptococcus pyogenes*	2 (1)	15 (1)
Cytomegalovirus	2 (1)	0 (0)
*Candida albicans*	2 (1)	0 (0)
Adenovirus	1 (<1)	9 (1)
*Bacteroides* spp	1 (<1)	3 (<1)
*Enterobacter* spp	1 (<1)	20 (1)
*Moraxella catarrhalis*	1 (<1)	13 (1)
*Mycobacterium tuberculosis*	1 (<1)	2 (<1)
*Pneumocystis jirovecii*	1 (<1)	0 (0)
Respiratory syncytial virus	1 (<1)	35 (2)
*Salmonella* spp	1 (<1)	1 (<1)
Other pathogens^b^	0 (0)	76 (5)

Abbreviation: ICH, immunocompromised host.

^a^Organisms are not mutually exclusive, and patients may have >1 organism identified.

^b^Other pathogens identified among non-ICHs, from greatest to least, were *Proteus* spp, *Mycoplasma pneumoniae*, *Legionella* spp, *Serratia* spp, *Citrobacter* spp, Nontuberculosis *mycobacteria*, *Stenotrophomonas* spp, *Pseudomonas* non-*aeruginosa*, *Actinomyces* spp, *Pasteurella multocida*, *Chlamydia pneumoniae*, *Morganella* spp, *Nocardia* spp, *Pseudomonas pseudomallei*.

### Time to Clinical Stability and Length of Stay

**Figure 4.**
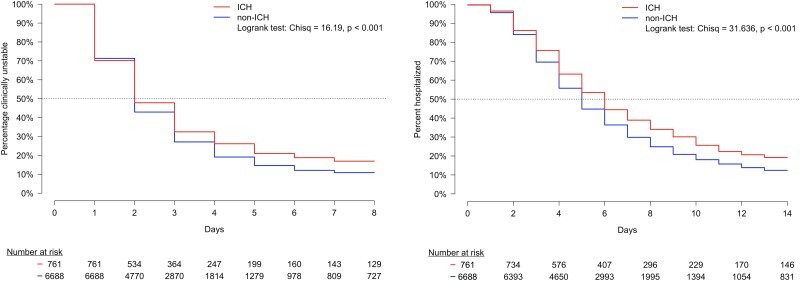
Time to event outcomes between ICHs and non-ICHs. *A*, Kaplan-Meier estimation and corresponding survival curves for time to clinical stability between ICHs and non-ICHs. Day 0 represents the first day of hospitalization, when all patients were clinically unstable. *B*, Kaplan-Meier estimation and corresponding survival curve for time to hospital discharge between ICHs and non-ICHs. Day 0 represents the first day of hospitalization, when all patients were still hospitalized. ICH, immunocompromised host.

Median time to clinical stability was 2 days for ICHs and 2 days for non-ICHs. After the second day, ICHs were less likely to become clinically stable (Figure 4*A*, *P* < .001). Median length of stay was 6 days for ICHs and 5 days for non-ICHs (Figure 4*B*, *P* < .001).

### Rehospitalization due to CAP

A total of 3544 patients were followed for rehospitalization due to CAP: 347 ICHs and 3197 non-ICHs. Among ICHs and non-ICHs, 16% patients in both groups were rehospitalized within 1 year (n = 54 vs n = 498, respectively; *P* > .999). For those rehospitalized, the median time to first rehospitalization for CAP was 151 days (IQR, 52–221) for ICHs and 123 days (IQR, 57–230) for non-ICHs (*P* = .938). There were no significant differences in cumulative incidence estimation between ICHs and non-ICHs (Supplementary Figure 2). Rehospitalization by immunocompromising condition is shown in Supplementary Figure 3.

### Mortality

**Figure 5.**
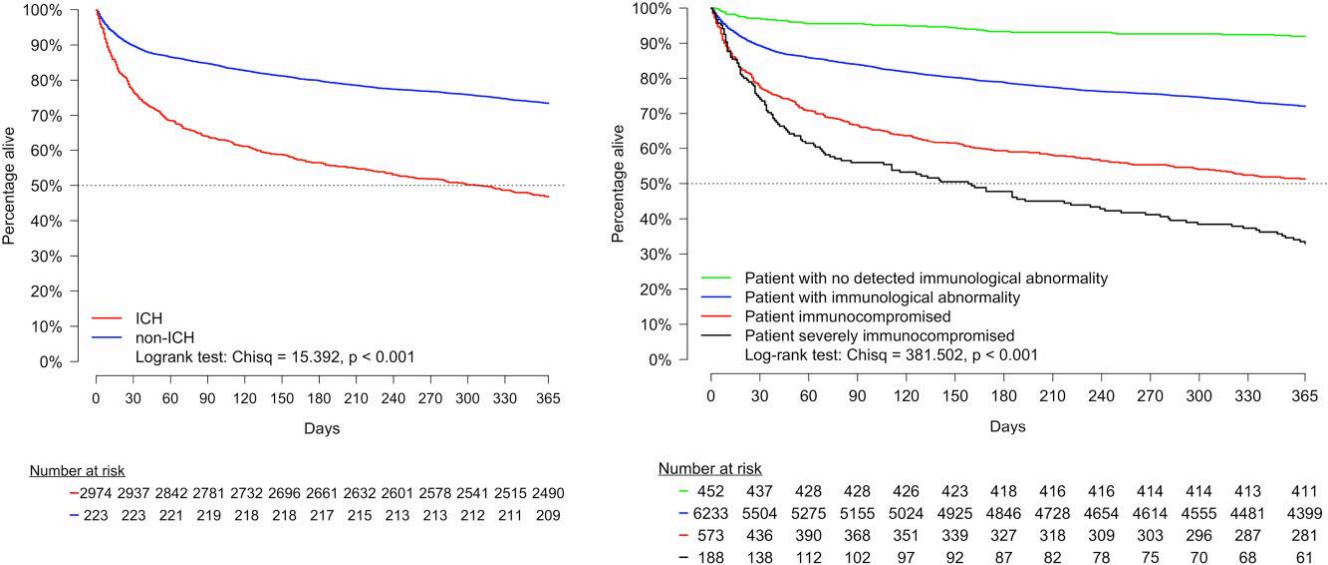
Time to mortality up to 1 year following hospital discharge. *A*, Time to mortality for ICHs and non-ICHs. *B*, Time to mortality for patients hospitalized with community-acquired pneumonia classified according to function of the immune system. ICH, immunocompromised host.

In-hospital mortality was 9% in ICHs and 5% in non-ICHs (*P* < .001). After hospitalization, all-cause mortality in ICHs vs non-ICHs was 24% vs 11% at 30 days, 44% vs 21% at 6 months, and 53% vs 27% at 1 year. Time to death from hospitalization for ICHs vs non-ICHs is depicted in Figure 5*A*. When patients were categorized according to function of the immune system, patients who were severely immunocompromised had the worst survival after hospitalization, followed by ones who were immunocompromised, those with an abnormal immune system, and patients without identifiable immunologic abnormality (Figure 5*B*). At 6 months, mortality for patients without identifiable immunologic abnormality was 7%; for those with abnormal immune function, 21%; for ones who were immunocompromised, 41%; and for patients who were severely immunocompromised, 51%. In the post hoc analysis, mortality within 1 year was 82% for ICHs with lung cancer, 50% for ICHs with corticosteroid use, and 20% advanced-stage HIV (see Supplementary Tables 6–8).

## DISCUSSION

We documented immunocompromising medical conditions or treatments in 10% of our cohort of hospitalized adults with CAP over the course of 2 years. In our study, we used CDC criteria to define a patient as an ICH. The same criteria are used by the National Institutes of Health and Infectious Diseases Society of America to define ICHs in the management of COVID-19 [19, 20]. The most common immunocompromising condition was advanced cancer, with lung cancer being the most common malignancy. A prior international multicenter study reported a point prevalence of 18% for ICHs hospitalized with CAP [21] and found chronic steroid use as the most frequent immunocompromising condition. The differences in findings when compared with our study may be explained by different designs as well as variability of ICHs among different countries.

Geospatial epidemiology suggests that areas in the city of Louisville with a high observed rate of ICHs hospitalized with CAP are associated with census tracts where a high proportion of individuals reside who have an income below the poverty level and are Black or African American. A link between hierarchies of social advantage and health has been described for multiple medical conditions in the United States [22]. Our data suggest that the risk for hospitalization for CAP in ICHs may be associated with socioeconomic and/or racial groups. Further studies evaluating the epidemiology of ICHs hospitalized with CAP should examine these disparity patterns, as well as other indicators of socioeconomic position, at smaller geographic units than the census tract.

Patients who were immunocompromised were hospitalized more frequently with high PSI scores as compared with patients who were nonimmunocompromised. In contrast, when evaluating patients using CURB-65, we found no statistically significant difference between the ICHs and non-ICHs. When we evaluated severity of disease within subgroups of ICHs, we identified cancer as the primary immunocompromising condition associated with severity of disease (Supplementary Table 3). One reason for this finding may be the number of patients with cancer in our cohort, as neoplastic disease is the second-most contributing factor to PSI score after age; for example, 2 patients could see as much as a 2-point difference in PSI risk class owing to the increase in score from neoplastic disease vs no disease. Scores such as the PSI and CURB-65 are frequently used to support clinical judgment in defining the need for hospitalization. Future studies are necessary to determine if PSI and/or CURB-65 has a role in supporting the need for hospitalization of patients who are immunocompromised.

An etiology of CAP was identified in 23% of ICHs and non-ICHs. In this series, the primary pathogen identified as the etiology of CAP in ICHs and non-ICHs was *S pneumoniae.* This was consistent with findings from 2 other studies [21, 23] indicating the relevance of *S pneumoniae* as the etiology of CAP in ICHs. In addition to *S pneumoniae*, methicillin-resistant *S aureus,* methicillin-sensitive *S aureus,* and *Pseudomonas aeruginosa* accounted for nearly two-thirds of identified etiologies in ICHs. The opportunistic pathogens identified in this series were *Aspergillus*, *Candida*, *Pneumocystis*, and *Cytomegalovirus*, accounting for 7% of identified etiologies in ICHs. Given the low number of identified pathogens, our findings should be interpreted with caution. Future studies with more comprehensive microbiological workup are necessary to define the etiology of CAP in ICHs.

The prevalence of any cardiovascular event was similar among ICHs and non-ICHs. The development of a new arrhythmia was the most common cardiovascular event in both groups.

During the first 2 days of hospitalization, the percentage of patients who reached clinical stability between ICHs and non-ICHs was similar. After 2 days, ICHs had delayed time to clinical stability. Since patients who are immunocompromised may lack fever or elevated white blood cell count at admission, our criteria for clinical stability should be interpreted with caution for this special population. The median length of stay in ICHs was 1 day longer when compared with non-ICHs. The prolonged hospitalization observed in ICHs may be due to a delay in clinical response. After hospitalization, both groups had a 16% rate of rehospitalization attributed to CAP, with a similar median time to rehospitalization (151 vs 123 days). When we evaluated rehospitalization within subgroups of ICHs, we identified advanced HIV infection as the primary immunocompromising condition associated with rehospitalization (Supplementary Figure 3).

Mortality for ICHs was almost doubled when compared with mortality in non-ICHs during hospitalization (9% vs 5%) and at 30 days (24% vs 11%), 6 months (44% vs 21%), and 1 year (53% vs 27%). Nearly 1 of every 2 ICHs hospitalized with CAP will die within 1 year after hospitalization. When patients were evaluated according to function of the immune system, we found that mortality increased as immune function decreased. At 6 months, mortality for patients without identifiable immunologic abnormality was 7%; for those with abnormal immune function, 22%; for patients who were immunocompromised, 40%; and for ones who were severely immunocompromised, 54%. In the post hoc analysis, ICHs with advanced lung cancer had the highest mortality rate, with nearly 4 of 5 patients dying within 1 year. In immunocompromised cases, CAP may be a marker of disease progression and play no role in long-term mortality for these patients. Further studies will be necessary to define if CAP accelerates underlying diseases and plays a role in the increased long-term mortality seen in these patients.

Our study has several limitations. Deterioration of a prior pulmonary infiltrate was a criterion for inclusion in our study. We cannot rule out that deterioration of the pulmonary infiltrate may have been primarily related to the progression of lung cancer, possibly producing an overestimation of this immunocompromising condition. Another limitation is that we used only medical history to define the level of immune function. In hospitalized patients with CAP, it will be important to define biological markers to have a more objective evaluation of the host immune system at the time of hospitalization. In our spatial epidemiology analysis, we relied on census tract–level aggregation of poverty, race, and age to compare the rates of ICHs hospitalized due to CAP, which limits our conclusions at the patient level; however, it provides key insights from a public health perspective at the community level. Further studies could improve on this analysis by collecting income and other socioeconomic factors at the patient level. In addition, we were unable to adjust for important confounders, such as social history and access to care, as these are not available at a census tract level of aggregation. Future studies should also assess if socioeconomic factors are associated with particular immunocompromising conditions.

Our study has several strengths. First, we were able to evaluate all adult hospitalizations in the city of Louisville for 2 consecutive years. Second, we were able to identify cases of CAP that were also included in our defined geographic area through the US Census Bureau using the patients’ home addresses. Third, we were able to define the number of unique patients hospitalized with CAP using social security numbers.

## CONCLUSION

In conclusion, approximately 10% of hospitalized patients with CAP are immunocompromised. Socioeconomic conditions may influence the risk for hospitalization of ICHs. We found an association between decreasing immune function and worsening clinical outcomes. There is a 2-fold increase in mortality at every time point for ICHs as compared with non-ICHs who are hospitalized due to CAP. Over half of ICHs died within 1 year after hospitalization, with mortality driven primarily by advanced lung cancer.

## Supplementary Material

ofad565_Supplementary_Data

## Supplementary Data

Supplementary materials are available at *Open Forum Infectious Diseases* online. Consisting of data provided by the authors to benefit the reader, the posted materials are not copyedited and are the sole responsibility of the authors, so questions or comments should be addressed to the corresponding author.

## Notes

***Acknowledgments***. We thank the Louisville CAP in the Immunocompromised Study Group for its assistance with the study. Members of the Louisville CAP in the Immunocompromised Study Group are as follows: Mohammed Khalid Abdulaziz Abbas, Ahmed Abdelhaleem, Aisha Olanike Adigun, Usman Ali Akbar, Oluwasegun Akinti, Ahmed Ali, Raghava Sekhar Ambadapoodi, Javaria Anwer, Saman Bahram, Aditya Bamboria, Laxman Bhandari, FNU Deepti, Joanna Ekabua, Sheref Abdelgawad Hassan Elseidy, Aiman Fatima, Farwah Fatima, Shivam Gulati, Syed Hassan, Shriya Khurana, Shameera Masthan, Rehab Salah Mohamed, Vivek Soorya Sathya Moorthy, Amal Mumtaz, Aleena Naeem, Keerthi Poladi, Lucia Puga Sanchez, Adnan Qureshi, Prasanna Raut, Vidyulata Salunkhe, Harideep Samanapally, Balaji Srinivasa Sekaran, Syed Zain Shah, Daniya Sheikh, Mohammad Tahboub, Rupalakshmi Vijayan, Mounica Vorla, Sudeep Yadav, and Zarlakhta Zamani.

***Potential conflicts of interest*.** All authors: No reported conflicts.
