# Supplementary material for: Community-Acquired Pneumonia in the Immunocompromised Host: Epidemiology and Outcomes
Source: Open Forum Infect Dis. 2023 Nov 22;10(11):ofad565. doi: 10.1093/ofid/ofad565 (PMC10676121; doi:10.1093/ofid/ofad565)
Supplement: ofad565_Supplementary_Data [file ofad565_supplementary_data.docx]

**Supplementary Materials**

**Community-Acquired Pneumonia in the Immunocompromised Host: Epidemiology and Outcomes**

| **Section Number** | **Section Title** | **Page Number** |
| --- | --- | --- |
| 1 | Participating hospitals of the parent study | 1 |
| 2 | Screening criteria | 4 |
| 3 | Immunocompromising conditions | 5 |
| 4 | Geospatial methods | 6 |
| 5 | Data quality | 8 |
| 6 | Supplemental tables and figures | 9 |

**1. Participating hospitals of the parent study**

From 2014-2016, a total of nine hospitals served the adult population of Louisville, Kentucky. All nine facilities were included in the University of Louisville Pneumonia Study, which was approved by each hospital prior to enrolling patients. Information regarding each participating institution is as follows:

*A. University of Louisville Hospital*

The University of Louisville Hospital evolved from a county hospital that started in 1823 with 150 beds to a state-of-the-art complex offering a complete range of inpatient and outpatient services. It is the primary adult teaching hospital for the University of Louisville's Health Sciences Center. With 404 licensed beds, the tertiary acute-care hospital provides a full range of diagnostic, therapeutic, emergency, and surgical services, including the region's only Level 1 adult trauma center.

*B. Robley Rex VA Medical Center*

The Robley Rex Veterans Administration (VA) Medical Center is a 120-bed acute care hospital located in Louisville, Kentucky. The VA hospital is one of the primary teaching hospitals of the University of Louisville Medical School.

*C. Baptist Healthcare Hospital*

With Joint Commission accreditation, this facility has specialized services for women's health, cancer, heart, orthopedics, neurosciences, emergency care, rehabilitation, sleep disorders, occupational health, and behavioral health, including psychiatric and chemical dependency care. This facility is licensed for 407 beds.

*D. Norton Audubon Hospital*

Norton Audubon Hospital offers a full range of highly specialized medical and surgical services, including an internationally recognized heart institute and newly expanded emergency department. This facility is licensed for 432 beds. Pneumonia is within their top 10 most common diagnoses.

*E. Norton Suburban Hospital*

Norton Suburban Hospital offers more than 1,200 physicians to patients in and around Louisville. Norton Suburban Hospital offers a full range of diagnostic, therapeutic, emergency and surgical services and is licensed for 343 beds.

*F. Norton Downtown Hospital*

Norton Hospital Downtown is focused on specialty surgical services, advanced diagnostics, oncology, orthopedics and maternal-fetal medicine. The hospital has served as a teaching facility for the University of Louisville School of Medicine for over 50 years. This facility is licensed for 955 beds and has all private rooms, higher cancer survival rates than national average, and advanced diagnostics and surgical technology.

*G. Jewish Hospital*

Jewish Hospital is a 442- bed tertiary medical center, which encompasses virtually every aspect of healthcare. Jewish Hospital is one of the premier heart hospitals in the U.S., dedicated to excellence in clinical care, research and education, and is a leader in Kentucky, being one of the first to achieve Chest Pain Accreditation. Jewish Hospital has been the site of many “firsts,” including the world’s first and second successful AbioCor® artificial heart implants and the world’s first hand transplant. Jewish Hospital is also federally designated to perform all five solid organ transplants–heart, lung, liver, kidney and pancreas.

*H. Saints Mary and Elizabeth Hospital*

Saints Mary and Elizabeth Hospital is a 298-bed facility. It is Louisville’s premier Catholic care giving facility. This facility offers many services, including surgical services, a state-of-the-art wound healing center, diagnostic imaging, cancer care, outpatient chemotherapy, cardiac services and a 20-bed emergency room.

*I. Norton Brownsboro Hospital*

Norton Brownsboro Hospital is Louisville’s newest hospital and offers a full range of inpatient and outpatient cancer services; cardiovascular and pulmonary services; neuroscience services; orthopedics and spine services; women’s services; and additional services such as emergency medicine, endoscopy, outpatient diagnostic services, physical/speech/occupational therapy and surgical services.

**2. Screening criteria**

All adult patients hospitalized were pre-screened for participation in the study using the following criteria: a chief complaint of cough, shortness of breath, shortness of air, fever, chills, malaise, or chest pain, or an admitting diagnosis of community-acquired pneumonia, pneumonia, altered mental status, confusion, fall, seizures, aspiration, cerebrovascular disease, transient ischemic attack, leukocytosis, leucopenia, respiratory failure, congestive heart failure, or urinary tract infection. Patients with at least one positive pre-screening criterion were screened. The screening criteria included a presence of a pulmonary infiltrate at chest x-ray or computed tomography scan of the chest. Patients with a pulmonary infiltrate were evaluated for inclusion into the study.

Inclusion criteria for the study were as follows: (1) presence of a new pulmonary infiltrate on chest radiograph and/or chest computed tomography scan at the time of hospitalization, defined by a board-certified radiologist’s reading; (2) at least 1 of the following: (a) new cough or increased cough or sputum production, (b) fever >37.8°C (100.0°F) or hypothermia <35.6°C (96.0°F), (c) changes in leukocyte count (leukocytosis: >11000 cells/μL; left shift: >10% band forms/mL; or leukopenia: <4000 cells/μL); and (3) no alternative diagnosis at the time of hospital discharge that justified the presence of criteria 1 and 2.

**3. Immunocompromising conditions**

1. *Immunocompromising conditions*

The following illnesses or medical treatments were used to define a patient as immunocompromised:

1. Primary immunodeficiency disease
2. Advanced stage cancer (stage III or IV cancer or hematologic cancer)
3. Advanced HIV infection (CD4 T-lymphocyte count <200 cells/mL or <14%)
4. Solid organ transplantation
5. Hematopoietic stem cell transplantation
6. Receiving cancer chemotherapy
7. Receiving biological immune modulators
8. Receiving corticosteroid therapy with a dose ≥20mg prednisone or equivalent daily for at least 14 days prior to hospitalization
9. Receiving disease-modifying antirheumatic drugs (DMARDs)
10. *Cancer staging definitions*

Cancer stages were defined as follows:

1. **Stage I**: Cancer localized to the organ or tissue of origin that has not spread to nearby lymph nodes or distant sites.
2. **Stage II**: Cancer that has progressed beyond the primary site and may have invaded nearby tissues or structures, but it has not yet metastasized to the lymph nodes or other parts of the body.
3. **Stage III**: Cancer that has invaded nearby lymph nodes and may have spread to adjacent tissues or organs, but it has not metastasized to distant sites.
4. **Stage IV**: Metastatic cancer.

**4. Geospatial methods**

The home address of each patient enrolled in the study was collected. An approximate latitude and longitude of the residence was obtained through the U.S. Census Bureau website which geomasked addresses through their geocoder tool. We used the census tract level for geospatial methods. Census tracts are subdivision of the U.S. Census Bureau data that account for a similar number of individuals with relative homogeneous socioeconomic indicators. Geospatial analyses were conducted using ArcGIS v10.2.

Using the latitude and longitude, we were able to aggregate the number of ICHs per census tract and report census-tract specific rates of ICHs hospitalized with CAP using the adult population as the denominator. A Local indicators of spatial association (LISA) map, using inverse distance weights, was produced to identify clusters of ICHs hospitalized due to CAP is depicted as **Supplementary Figure 1**, below.


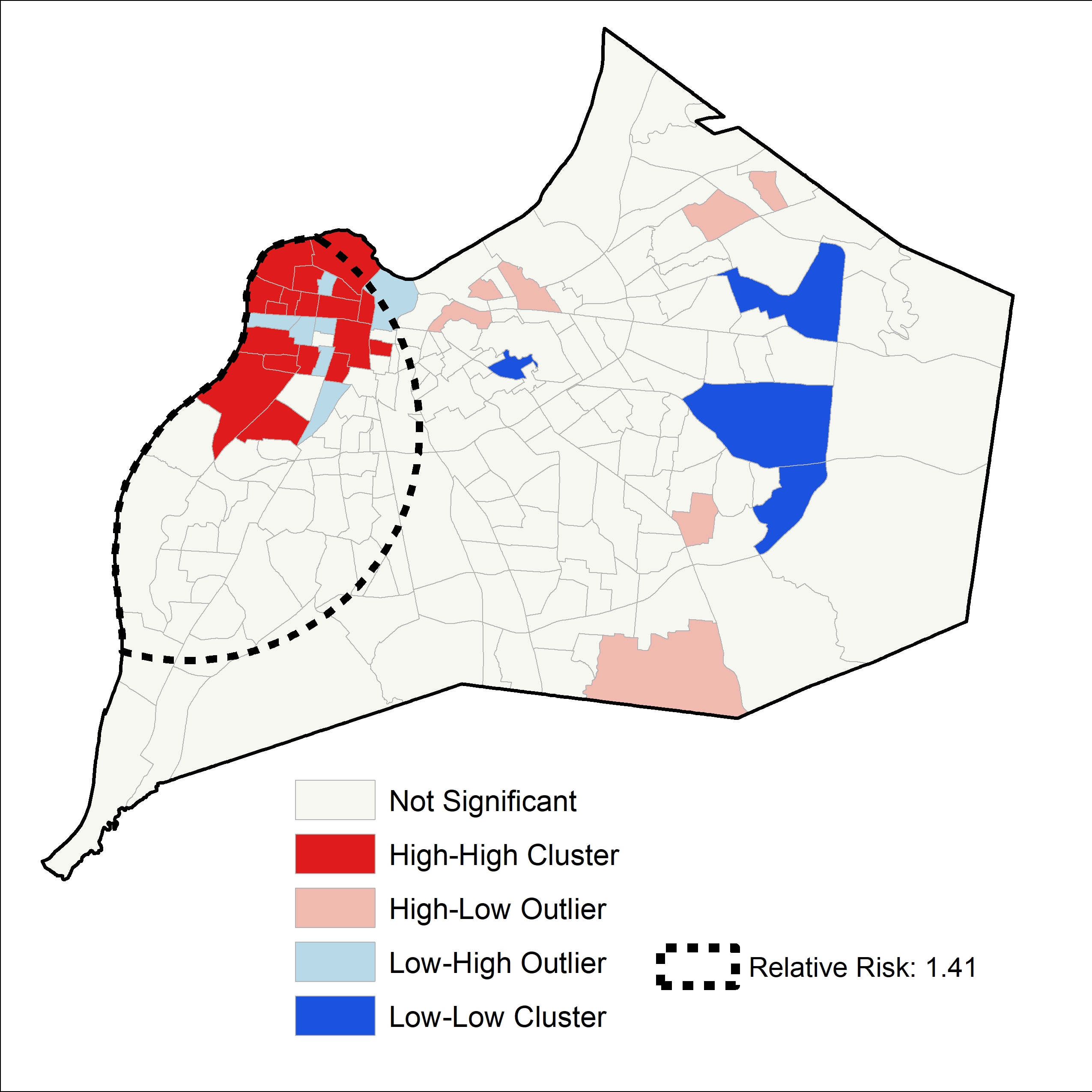


**Supplementary Figure 1. LISA map depicting rates of ICH CAP.**

Clusters of high-rate census tracts are colored in the dark red and clusters of low rates are colored in dark blue. Light red and blue colored census tracts depict outliers, where the census tract had a high or low rate in comparison to the neighboring census tract, respectively.

A heatmap was created using location of each unique patient’s home address at the time of first hospitalization each study year. Heat maps of Louisville were developed using census tract-level data representing the distribution of income, Black/African American, and elderly populations by census tract. To create heat maps this way, census tract centroids were used, with a population field equal to the number of individuals of the category, and a computed density area of 2 miles. Areas of high risk of hospitalization due to CAP were calculated using Kulldorff’s Spatial Scan Statistic^[[1]](#footnote-1)^. Using SaTScan software, we used a purely spatial Poisson model without covariates. Census tract data were imported into SaTScan, with cases defined as census tract aggregates of ICHs hospitalized due to CAP, aggregates of the adult population per census tract, with longitude and latitude of census tract centroids to determine location.

**6. Data quality**

Trained study coordinators and/or research associates collected clinical data from the patient’s medical record onto a paper case report form. A separate research associate entered these data into a secure, web-based data management system hosted by the University of Louisville Division of Infectious Diseases. Data quality issues identified by the research associate entering the data were fixed prior to submission of the case to the database. The database also contains several data quality checks to limit out-of-range errors and inappropriate data types. These data quality structures were built in based on our decades of experience collecting and entering clinical data into electronic databases. After all data queries were resolved, the case was accepted into the database for analysis.

**7. Supplemental tables and figures**

**Supplementary Table 1. Description of ICHs with more than one medical condition or treatment**

|  | Advanced-stage cancer | Cancer Chemotherapy | Corticosteroid therapy | DMARDS | Advanced HIV infection | Solid organ transplantation | Biological Immune Modulator | Primary immunodeficiency | Hematopoietic stem cell transplant |
| --- | --- | --- | --- | --- | --- | --- | --- | --- | --- |
| Advanced-stage cancer, n=400 |  | 32% | 9% | 1% | 1% | 1% | 1% | 1% | 1% |
| Cancer Chemotherapy, n=178 | 71% |  | 15% | 1% | 1% | 0% | 3% | 1% | 2% |
| Corticosteroid therapy, n=153 | 24% | 17% |  | 5% | 1% | 1% | 2% | 1% | 1% |
| DMARDS, n=76 | 4% | 1% | 11% |  | 0% | 3% | 5% | 1% | 0% |
| Advanced HIV infection, n=50 | 6% | 2% | 4% | 0% |  | 0% | 0% | 0% | 0% |
| Solid organ transplantation, n=42 | 5% | 0% | 5% | 5% | 0% |  | 2% | 0% | 0% |
| Biological Immune Modulator, n=36 | 11% | 14% | 8% | 11% | 0% | 3% |  | 0% | 0% |
| Primary immunodeficiency, n=32 | 6% | 6% | 6% | 3% | 0% | 0% | 0% |  | 3% |
| Hematopoietic stem cell transplant, n=11 | 45% | 36% | 9% | 0% | 0% | 0% | 0% | 9% |  |

Percentages depict the percentage of a row element that is shared by the column. (e.g. 71% of cancer chemotherapy patients had advanced stage cancer)**Supplementary Table 2. Physical exam findings, laboratory values, and initial severity of disease**

|  | ICH | non-ICH | p-value |
| --- | --- | --- | --- |
|  | 761 | 6688 |  |
| Heart Rate (beats/min), median [IQR] | 110 [96, 123] | 105 [91, 119] | <0.001 |
| Respiratory Rate (breaths/min), median [IQR] | 22 [20, 27] | 22 [20, 27] | 0.346 |
| Respiratory rate > 30 breaths/min, n (%) | 128 (17) | 1128 (17) | >0.999 |
| Systolic Blood Pressure (SBP) (mmHg), median [IQR] | 109 [96, 127] | 116 [100, 135] | <0.001 |
| Diastolic Blood Pressure (DBP) (mmHg), median [IQR] | 55 [48, 64] | 57 [49, 68] | 0.001 |
| SBP < 90 mmHG or DBP <60 mmHg, n (%) | 471 (62) | 3673 (55) | <0.001 |
| Temperature (degrees C), median [IQR] | 37.2 [36.8, 37.8] | 37.2 [36.8, 37.9] | 0.618 |
| Temperature < 36 degrees C, n (%) | 6 (1) | 123 (2) | 0.050 |
| O2 saturation (%), median [IQR] | 94 [91, 96] | 94 [91, 96] | 0.151 |
| WBC x 1000 per uL, median [IQR] | 11.3 [6.8, 15.8] | 12.3 [8.8, 16.6] | <0.001 |
| WBC < 4000 cells/uL, n (%) | 103 (14) | 240 (4) | <0.001 |
| Platelet count x 1000 per uL, median [IQR] | 208 [151, 285] | 224 [172, 289] | <0.001 |
| Platelet count < 100,000 cells/uL, n (%) | 92 (12) | 327 (5) | <0.001 |
| Hematocrit (%), median [IQR]) | 32.8 [28.6, 36.7] | 36.0 [31.9, 40.1] | <0.001 |
| Glucose (mg/dL), median [IQR]) | 138 [111, 185] | 143 [114, 197] | 0.022 |
| Blood urea nitrogen (mg/dL), median [IQR]) | 18 [13, 28] | 19 [13, 29] | 0.253 |
| Blood urea nitrogen > 20 mg/dL, n (%) | 356 (47) | 3276 (49) | 0.265 |
| Creatinine (mg/dL), median [IQR]) | 0.9 [0.7, 1.4] | 1.0 [0.8, 1.4] | <0.001 |
| Arterial blood gasses obtained, n (%) | 273 (36.2) | 2680 (40.0) | 0.033 |
| pH, median [IQR] | 7.4 [7.4, 7.4] | 7.4 [7.3, 7.4] | 0.013 |
| PaCO2 (mmHg), median [IQR] | 38.8 [33.7, 49.0] | 41.0 [34.4, 51.0] | 0.066 |
| PaO2 (mmHg), median [IQR] | 74.0 [62.0, 93.3] | 72.0 [61.0, 92.0] | 0.640 |
| Bicarbonate (mEq/L), median [IQR] | 25.0 [22.0, 29.0] | 25.0 [22.0, 29.0] | 0.932 |
| FiO2 (%), median [IQR] | 36.0 [28.0, 52.0] | 36.0 [28.0, 60.0] | 0.534 |
| PaO2/FiO2 ratio < 250, n (%) | 164 (60) * | 1684 (62) * | 0.405 |
| Multilobar infiltrates, n (%) | 327 (43) | 2786 (42) | 0.511 |
| Confusion / disorientation, n (%) | 112 (15) | 1295 (19) | 0.002 |
| Need for invasive mechanical ventilation, n (%) | 38 (5) | 391 (6) | 0.382 |
| Need for vasopressors, n (%) | 19 (2) | 194 (3) | 0.604 |
| *Percentage is out of total with arterial blood gasses obtained | | | |

**Supplementary Table 3. Pneumonia severity scores by immunocompromising conditions**

|  | Advanced-stage cancer | Advanced HIV infection | Cancer chemotherapy | Corticosteroid therapy | DMARDs | Other* |
| --- | --- | --- | --- | --- | --- | --- |
| ***Overall***** | ***N = 400*** | ***N = 50*** | ***N = 178*** | ***N = 153*** | ***N = 76*** | ***N = 119*** |
| PSI Class IV or V | 364 (91%) | 15 (30%) | 160 (90%) | 105 (69%) | 43 (57%) | 68 (57%) |
| CURB-65 3, 4, or 5 | 140 (35%) | 8 (16%) | 61 (34%) | 56 (37%) | 33 (43%) | 28 (24%) |

*Other includes patients with a history of either solid or hematopoietic transplantation, primary immune deficiency, or use of biologics.

**Categories are not mutually exclusive and patients may be represented more than once

**Supplementary Table 4. Rates of in-hospital cardiovascular events between ICH vs non-ICH**

|  | ICH | non-ICH | p-value |
| --- | --- | --- | --- |
| n | 761 | 6688 |  |
| Any event (%) * | 47 (6.2) | 520 (7.8) | 0.133 |
| New arrhythmia (%) | 31 (4.1) | 218 (3.3) | 0.283 |
| Worsening of existing arrhythmia (%) | 11 (1.5) | 150 (2.2) | 0.191 |
| Pulmonary edema (%) | 1 (0.1) | 39 (0.6) | 0.175 |
| Acute MI (%) | 4 (0.5) | 127 (1.9) | 0.010 |
| Pulmonary embolism (%) | 1 (0.1) | 35 (0.5) | 0.230 |
| Stroke (%) | 1 (0.1) | 16 (0.2) | 0.848 |

*Cardiovascular events are not exclusive and some patients may have experienced multiple cardiovascular events during hospitalization

**Supplementary Table 5. Standard of care specimen obtained among ICH and non-ICH patients hospitalized due to CAP**

|  | ICH | non-ICH | p-value |
| --- | --- | --- | --- |
| n | 761 | 6688 |  |
| Blood culture (%) | 698 (91) | 6025 (90) | 0.573 |
| Respiratory culture (%) | 273 (36) | 2193 (33) | 0.094 |
| Pneumococcal urinary antigen test (%) | 117 (15) | 1165 (17) | 0.172 |
| *Legionella* urinary antigen test (%) | 109 (14) | 1087 (16) | 0.186 |
| Rapid Influenza test (%) | 131 (17) | 1422 (21) | 0.011 |
| Viral RT-PCR test (%) | 141 (19) | 1066 (16) | 0.074 |
| Atypical pathogens PCR test (%) | 98 (13) | 860 (13) | >0.999 |

**Supplementary Figure 2. Cumulative incidence of time to rehospitalization**


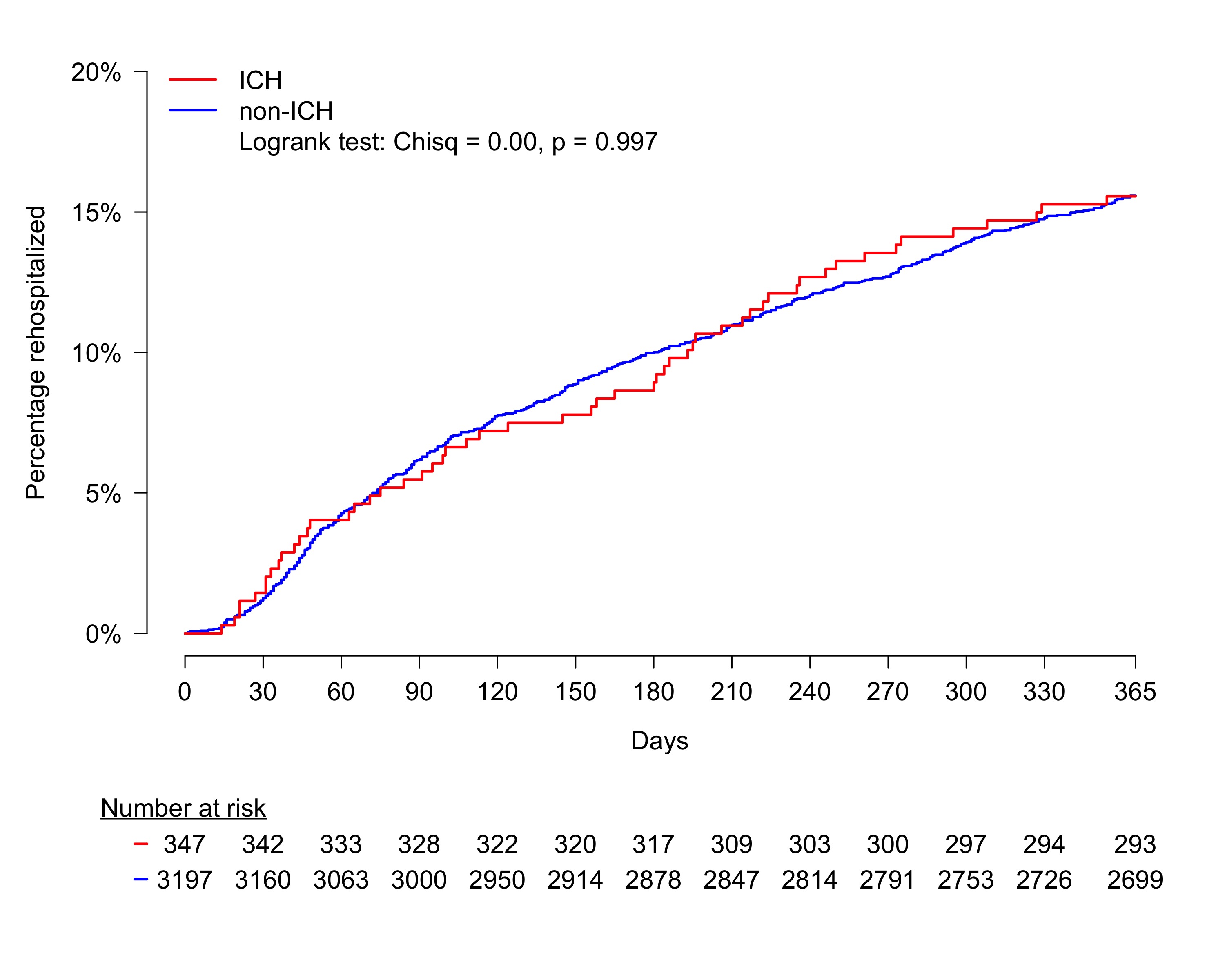


**Supplementary Figure 3. One-year rehospitalization rates by immunocompromising condition**


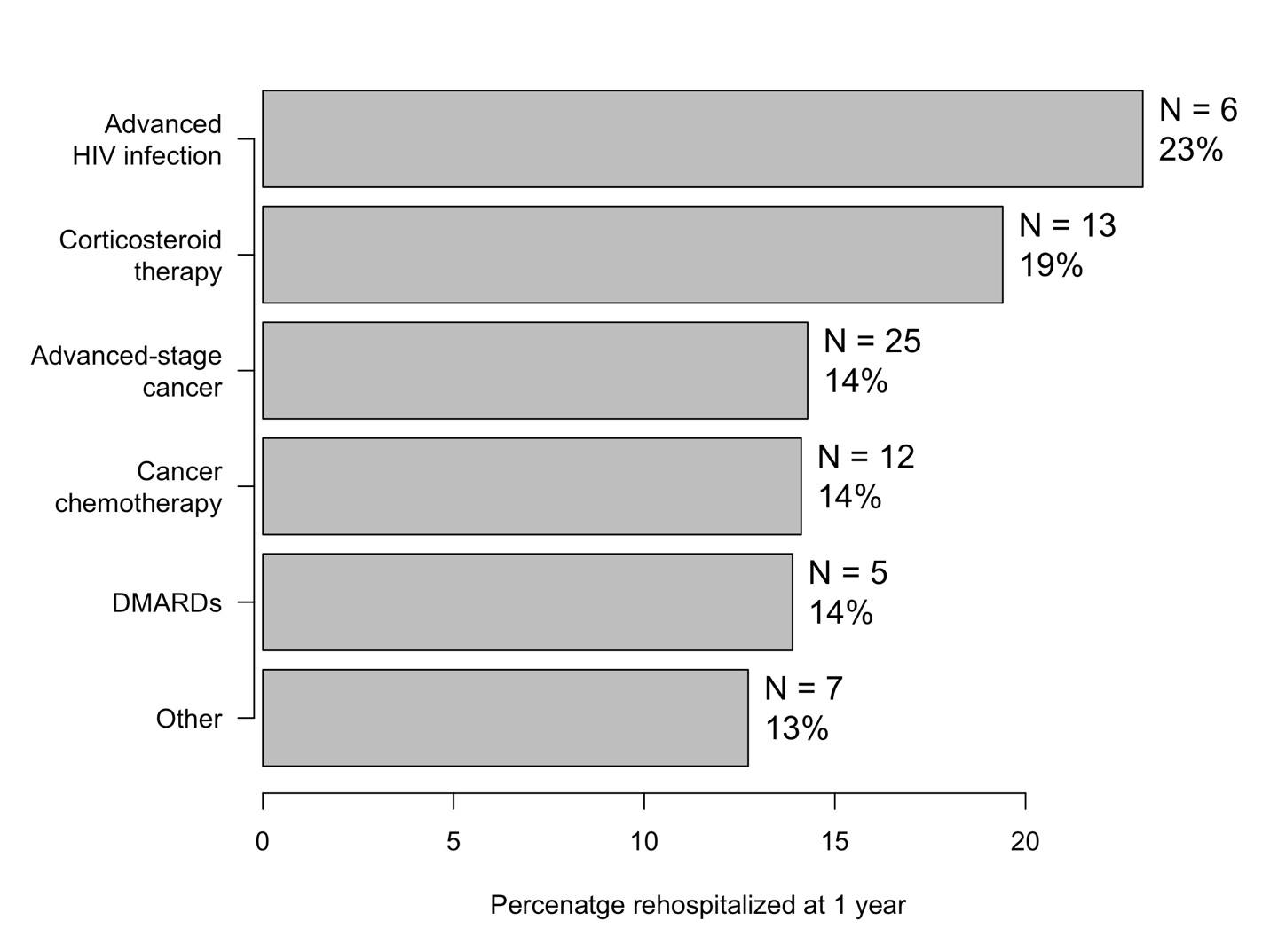


*Other includes patients with a history of either solid or hematopoietic transplantation, primary immune deficiency, or use of biologics.

**Supplementary Table 6. Post-Hoc Analysis of Outcomes: Advanced Stage Lung Cancer vs. Other Immunocompromising Conditions vs. Non-Immunocompromised**

|  | Advanced-stage  lung cancer | Other ICH | Non-ICH | p-value |
| --- | --- | --- | --- | --- |
| N | 181 | 580 | 6688 |  |
| In-hospital cardiac event (%) | 13 (7.2) | 34 (5.9) | 520 (7.8) | 0.243 |
| TCS (censored) (median [IQR]) | 3 [1, 7] | 2 [1, 4] | 2 [1, 4] | 0.007 |
| LOS (censored) (median [IQR]) | 7 [4, 13] | 6 [3, 10] | 5 [3, 8] | <0.001 |
| In-hospital mortality (%) | 29 (16.0) | 44 (7.6) | 356 (5.3) | <0.001 |
| 30 day mortality (%) | 65 (36.5) | 115 (20.3) | 720 (10.9) | <0.001 |
| 6 month mortality (%) | 120 (67.4) | 204 (36.5) | 1349 (20.5) | <0.001 |
| 1 year mortality (%) | 146 (82.0) | 248 (44.5) | 1779 (27.1) | <0.001 |

**Supplementary Table 7. Post-Hoc Analysis of Outcomes: Corticosteroid use vs. Other Immunocompromising Conditions vs. Non-Immunocompromised**

|  | Corticosteroid use | Other ICH | Non-ICH | p-value |
| --- | --- | --- | --- | --- |
| N | 153 | 608 | 6688 |  |
| In-hospital cardiac event (%) | 14 (9.2) | 33 (5.4) | 520 (7.8) | 0.087 |
| TCS (censored) (median [IQR]) | 2 [1, 4] | 2 [1, 5] | 2 [1, 4] | 0.044 |
| LOS (censored) (median [IQR]) | 5 [4, 9] | 6 [4, 11] | 5 [3, 8] | <0.001 |
| In-hospital mortality (%) | 10 (6.5) | 63 (10.4) | 356 (53) | <0.001 |
| 30 day mortality (%) | 25 (16.6) | 155 (26.1) | 720 (10.9) | <0.001 |
| 6 month mortality (%) | 57 (38.3) | 267 (45.4) | 1349 (20.5) | <0.001 |
| 1 year mortality (%) | 75 (50.3) | 319 (54.4) | 1779 (27.1) | <0.001 |

**Supplementary Table 8. Post-Hoc Analysis of Outcomes: Advanced HIV infection vs. Other Immunocompromising Conditions vs. Non-Immunocompromised**

|  | Advanced HIV infection | Other ICH | Non-ICH | p-value |
| --- | --- | --- | --- | --- |
| N | 50 | 711 | 6688 |  |
| In-hospital cardiac event (%) | 1 (2.0) | 46 (6.5) | 520 (7.8) | 0.149 |
| TCS (censored) (median [IQR]) | 3 [1, 4] | 2 [1, 5] | 2 [1, 4] | 0.06 |
| LOS (censored) (median [IQR]) | 5 [3, 8] | 6 [4, 11] | 5 [3, 8] | <0.001 |
| In-hospital mortality (%) | 0 (0.0) | 73 (10.3) | 356 (5.3) | <0.001 |
| 30 day mortality (%) | 4 (8.0) | 176 (25.4) | 720 (10.9) | <0.001 |
| 6 month mortality (%) | 8 (16.0) | 316 (46.0) | 1349 (20.5) | <0.001 |
| 1 year mortality (%) | 10 (20.4) | 384 (56.0) | 1779 (27.1) | <0.001 |

1. Kulldorff M. A spatial scan statistic. *Communications in Statistics - Theory and Methods.* 1997;26(6):1481-1496. [↑](#footnote-ref-1)
